# Biological basis of critical illness subclasses: from the bedside to the bench and back again

**DOI:** 10.1186/s13054-024-04959-3

**Published:** 2024-05-29

**Authors:** Joseph Stevens, Oğuzhan Tezel, Valentina Bonnefil, Matthew Hapstack, Mihir R. Atreya

**Affiliations:** 1 Division of Immunobiology, Graduate Program, College of Medicine, University of Cincinnati, Cincinnati, OH 45267 USA; 2 Division of Critical Care Medicine, Cincinnati Children’s Hospital Medical Center, Cincinnati, OH 45229 USA; 3 Department of Pediatrics, University of Cincinnati College of Medicine, Cincinnati, OH 45627 USA

**Keywords:** Precision medicine, Critical illness subclass, Endotype, Phenotype

## Abstract

Critical illness syndromes including sepsis, acute respiratory distress syndrome, and acute kidney injury (AKI) are associated with high in-hospital mortality and long-term adverse health outcomes among survivors. Despite advancements in care, clinical and biological heterogeneity among patients continues to hamper identification of efficacious therapies. Precision medicine offers hope by identifying patient subclasses based on clinical, laboratory, biomarker and ‘omic’ data and potentially facilitating better alignment of interventions. Within the previous two decades, numerous studies have made strides in identifying gene-expression based endotypes and clinico-biomarker based phenotypes among critically ill patients associated with differential outcomes and responses to treatment. In this state-of-the-art review, we summarize the biological similarities and differences across the various subclassification schemes among critically ill patients. In addition, we highlight current translational gaps, the need for advanced scientific tools, human-relevant disease models, to gain a comprehensive understanding of the molecular mechanisms underlying critical illness subclasses.

**Graphical abstract:**

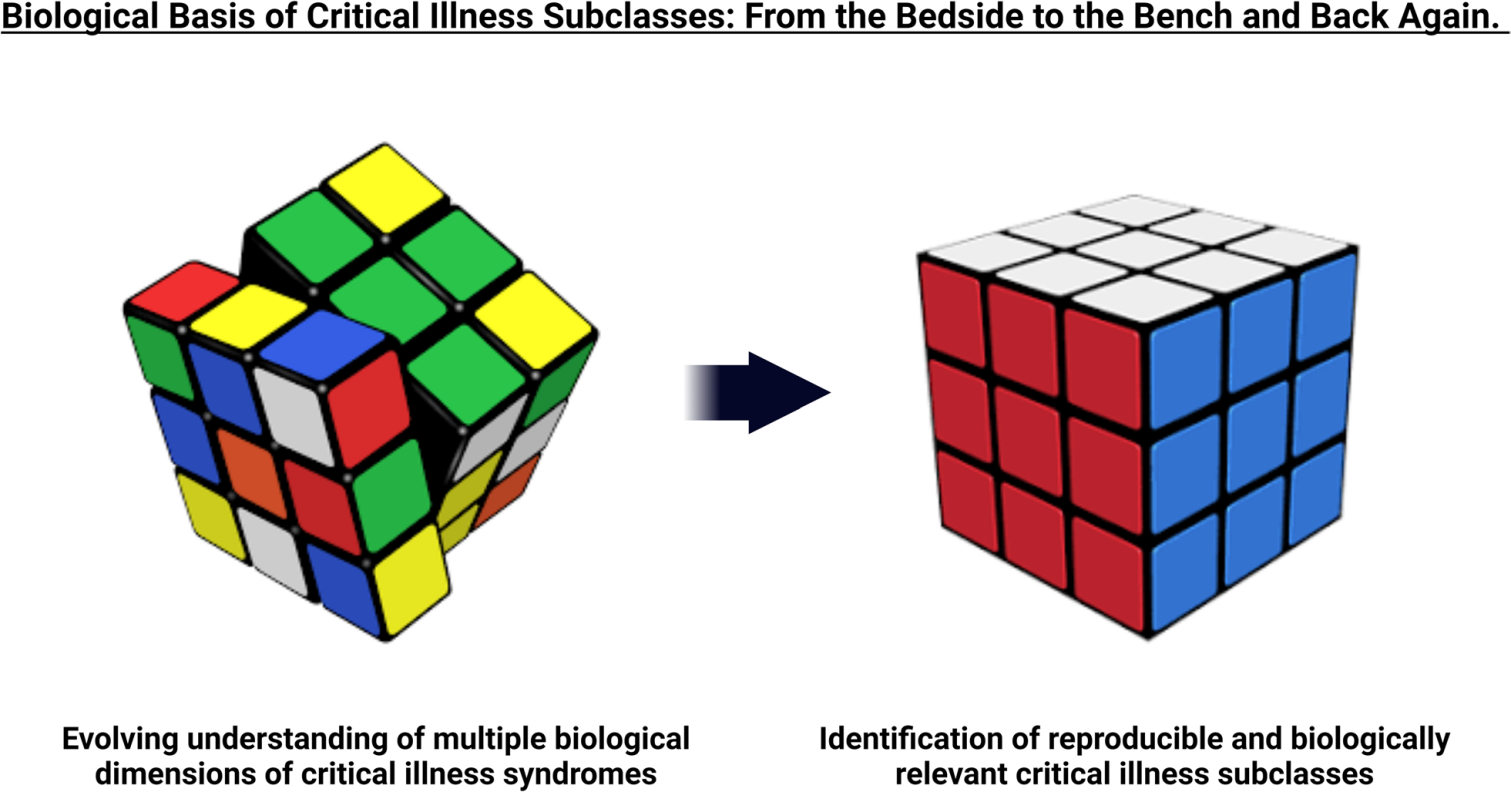

## Background

Critical illness syndromes including sepsis, acute respiratory distress syndrome (ARDS), and acute kidney injury (AKI) affect pediatric and adult patients admitted to intensive care units (ICUs) across the world [1–5, and are associated with high mortality. Moreover, survivors of critical illness syndromes remain at risk of long-term health consequences including chronic debilitation 6, technology dependence 7, and late death [8–10. Despite this burden of disease, care for such patients remains largely limited to antibiotics and intensive organ support. One-size-fits-all approaches to modulate the host response among critically ill patients have been met with repeated failure in clinical trials 11. This lack of efficacy of drugs has been attributed to administration of therapies in unselected populations and due to unaccounted variation among patients 12, 13. Thus, identification of the *right drug in the right patient at the right time* remains a major challenge.

## Era of precision critical care medicine

Precision medicine approaches seek to address these challenges by identifying subsets of critically ill patients based on shared features including clinical, laboratory, biomarker, or ‘omic’ data 13. Broadly, these include (1) prognostic enrichment tools that seek to identify groups of patients based on risk of outcomes such as mortality, and (2) predictive enrichment tools that seek to identify groups of patients based on shared biological pathways, which may be amenable to therapeutic intervention 14. While numerous promising subclassification schemes have emerged within the previous two decades, each with prognostic, predictive implications, or both, few have translated into the clinical realm. Moreover, we lack a comprehensive understanding of underlying disease mechanisms, a challenge further hampered by the limited translation of current disease models used to study critical illness pathobiology. Thus, much progress needs to be made in identifying *treatable traits* underlying critical illness subclasses. 13

In this state-of-the-art review, we summarize subclassification schemes that have demonstrated reproducibility in identifying biologically distinct subgroups of patients. While the relevance of critical illness subclasses have been previously considered elsewhere 15, 16, we seek to highlight key molecular similarities and differences across the subclassification schemes. Finally, we highlight current knowledge gaps, translational approaches, and disease models that may help augment our mechanistic understanding of subclass-specific biology and accelerate progress toward drug repurposing as well as the discovery of de novo targeted therapies.

## Evolving understanding of the host response in critical illness

**Fig. 1:**
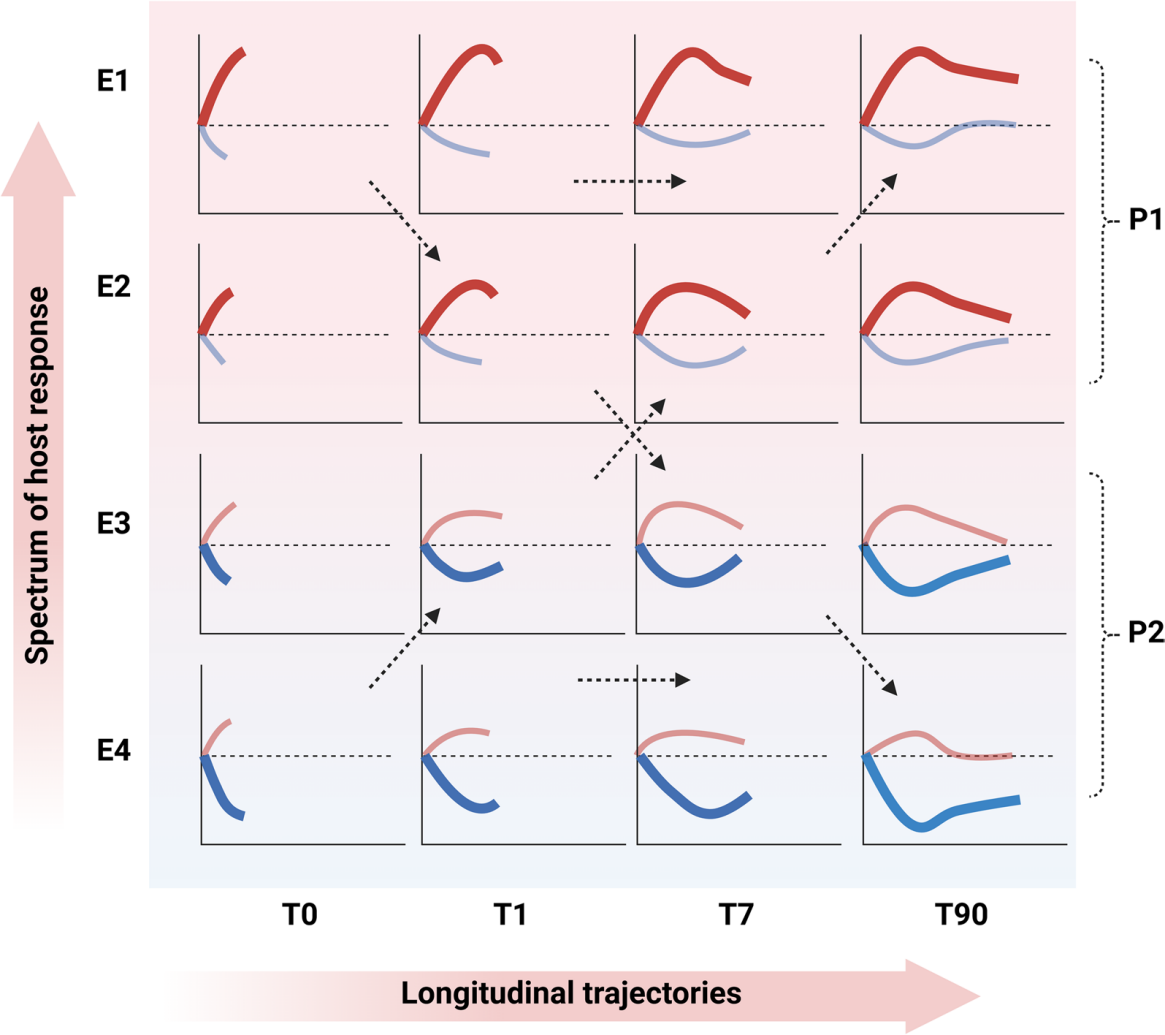
An evolving paradigm of dysfunctional host response in critical illness. Critically ill patients exhibit a spectrum of dysfunctional pro- and anti- inflammatory responses at illness onset, which are subject to change over the longitudinal course of critical illness influenced by host–pathogen-environment related factors. Each column of figures represent time points across the course of critical illness (T0-T90 in days). Each row (E1-4) represents critical illness endotypes. Viewed through an alternate lens, the top two rows together represent a *hyperinflammatory* phenotype (P1) and the bottom two rows together represent a *hypoinflammatory* phenotype (P2). The uppermost row represents patients characterized by overexpression of the innate immune response (red) and repression of the adaptive immune response (blue). Given the lack of negative feedback by the adaptive immune system, these patients continue to have sustained and unchecked hyperinflammation. On the other end of the spectrum, are patients with overactivation of anti-inflammatory pathways resulting in severely immunosuppressed state. Further, patients may exhibit temporal subclass switching over the course of critical illness, including those in response to treatments or interventions received

The classical paradigm of dysfunctional host responses among critically ill patients imply a sequential activation of the host innate and adaptive immune response resulting in a systemic inflammatory response syndrome (SIRS) followed by a compensatory anti-inflammatory response (CARS) syndrome 17, 18. However, it is increasingly understood that both the innate and adaptive arms of the host immune response may contribute to pro- and anti-inflammatory responses relatively early in the course of illness with significant crosstalk between them. An evolving paradigm of dysfunctional host response among those critically ill suggests that patients manifest a spectrum of maladaptive responses at illness onset that are subject to change over the course of disease under the influence of myriad pathogen-host-environment related factors. This concept is illustrated in Fig. 1.

**Fig. 2:**
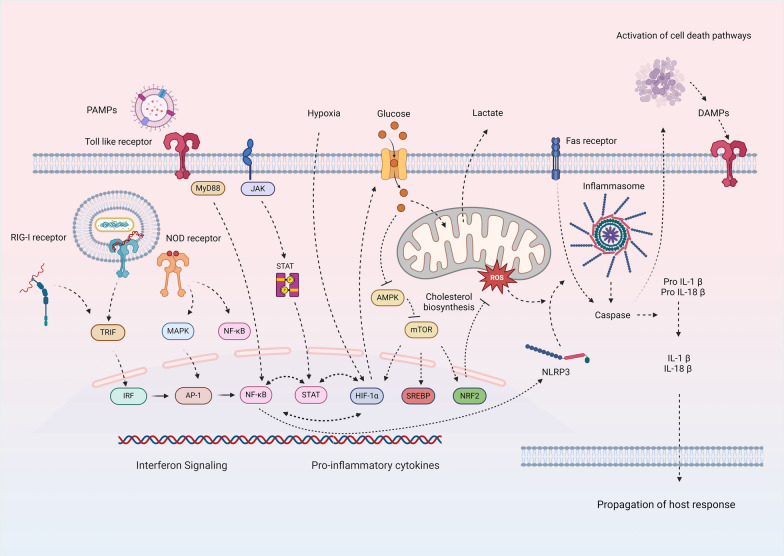
Intra-cellular signaling links immune response, metabolic state, and cellular fate. Pathogen recognition receptors (PRR) including toll like receptors (TLR), nucleotide oligomerization domain (NOD)-like receptors, and retinoic acid inducible gene-I-like receptors (RIG-I) detect extra- and intra-cellular pathogens. Activation of PRRs results in transcription of key pro-inflammatory pathways including nuclear factor kappa B (NFκB) and mitogen activated protein kinase (MAPK) signaling. This is facilitated by adaptor proteins including myeloid differentiation primary response 88 (MyD88) protein or toll-interleukin receptor domain–containing adaptor protein–inducing interferon-β (TRIF) – the latter being dominant in the host response to viral infections. Under hypoxic conditions, hypoxia inducible factor 1α (HIF1α) signaling triggers metabolic shift from oxidative phosphorylation towards glycolysis and cholesterol biosynthesis through mammalian target of rapamycin (mTOR) signaling. Although an efficient mechanism to maintain cellular function, a side effect of anerobic respiration is the production of reactive oxygen species (ROS). ROS induce a second hit and induce activation of inflammasome through nod like receptor pyrin domain 3 (NLRP3) protein resulting in activation of caspases. The latter serve to propagate the host response by cleaving protein precursors of inflammatory cytokines including interleukin-1 and 18 or serve to activate cell death pathways including apoptosis. Thus, the host immune response is inextricably linked to cellular metabolism and cellular fate. As detailed in the **Table** and illustrated in Fig. 4, although current subclassification schemes among critically ill patients sample the same set of key biological pathways they yield non-synonymous class outputs

**Fig. 3:**
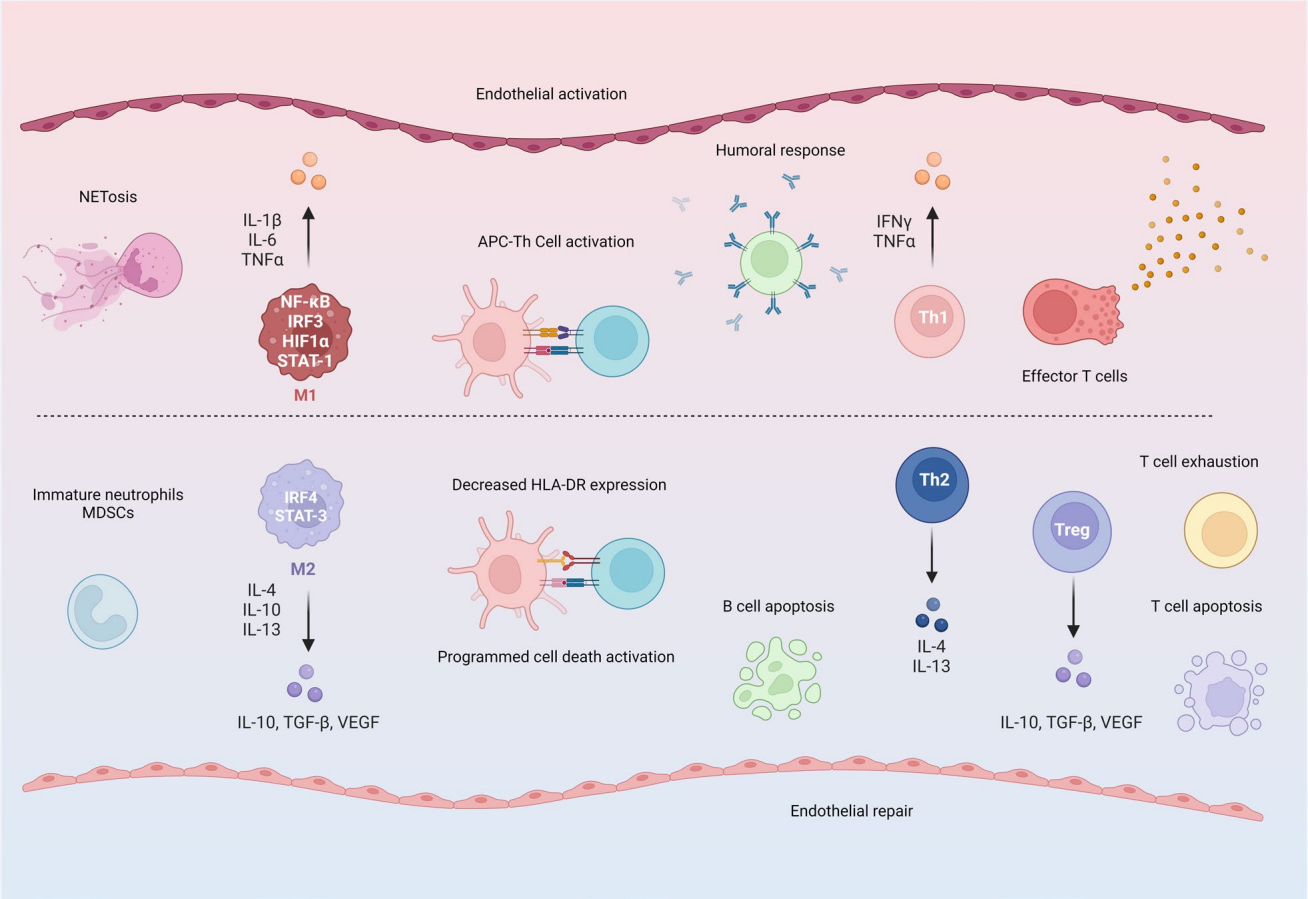
Summary of shifts in cellular states and landscape among critically ill patients. The left half of the image shows the innate arm while the right half shows the adaptive arm of the immune response. The top half of the figure shows molecular features that drive a pro-inflammatory state. Neutrophils release extracellular traps (NETs) resulting in NETosis that serve to facilitate phagocytosis of pathogens. Monocytes may be driven toward a pro-inflammatory M1 macrophage under the influence of NFκB, HIF1α, signal transducer and activator of transcription 1 (STAT1), and interferon regulatory factor 3 (IRF3). Innate immune antigen presenting cells (APC) engage T-helper (Th) cells through expression of human leukocyte antigen (HLA) DR. B-lymphocytes produce antibodies driving the humoral adaptive response. Th1 differentiation of helper cells results in secretion of pro-inflammatory cytokines including interferon gamma (IFNγ) and tumor necrosis factor alpha (TNFα) which further propagates the innate immune response. Finally, cytotoxic T cells release granzyme and result in cell death. The bottom half depicts features that drive an anti-inflammatory state in patients. There may be a shift towards immature innate cells including developing neutrophils and myeloid derived suppressor cells (MDSCs)– the early and late phases of critical illness, respectively. Monocytes may be polarized to an immunosuppressive M2 phase under the influence of interleukins -4, 10, and 13. There is decrease in HLA-DR expression and activation of co-stimulatory programmed cell death (PD-1L-PD-1) pathway. Differentiation towards a Th2 phenotype results in secretion of IL-4 and 13. Expansion of regulatory T cells result in IL-10, transforming growth factor beta (TGFβ) and vascular endothelial growth factor (VEGF) that are immunosuppressive and thought to promote tissue repair and remodeling. With prolonged illness, B- and T- lymphocytes may exhibit immune exhaustion with metabolic failure ultimately triggering cell death. As detailed in the **Table** and illustrated in Fig. 4, although current subclassification schemes among critically ill patients largely represent shifts across similar cellular states and landscape they yield non-synonymous class outputs

Recognition of pathogen- and damage- associated molecular patterns (PAMPs and DAMPs) initiate and propagate the host response in critical illness. Although not the primary objective of this review, we provide an overview of the perturbations in intra-cellular pathways linking inflammatory signaling, metabolic state, and cellular fate central to the dysregulated host response among critically ill patients in Fig. 2**.** Further, the shifts in immune states and cellular landscape among critically ill patients that contribute to pro- and anti-inflammatory responses are summarized in Fig. 3. Our objective here is to provide the reader the necessary context to understand the biological basis of critical illness subclasses discussed herein.

## Subclassification schemes in critical illness

It is worth noting that the subclassification schemes that have emerged sample various facets of the host response–the proverbial ‘biological pie’– in differing permutations and combinations and at varying depth. Given that gene-expression and clinico-biomarker models using peripheral blood have been most frequently utilized to identify critical illness subclasses and likely provide the most comprehensive understanding of biological mechanisms thus far, we have chosen to focus our attention on these approaches. In Table 1**,** we have compiled biological similarities and differences across numerous subclassification schemes among critically ill patients. Key studies have been summarized in the following paragraphs.

**Table 1:** Summary of critical illness subclassification schemes with gene-expression data used to comprehensively characterize biological basis of subclasses

Study, syndrome, population	Platform, approach, sample size	Subclass	Biological basis	Clinical relevance
1. Wong et al. 21	Whole blood derived RNA,	Subclass A, B, and C	Endotype A characterized by repression of the adaptive arm of immune system, T-cell function, and glucocorticoid signaling, relative to patients with endotype B	Endotype A was associated with a nearly threefold higher rate of mortality relative to other subclasses
Pediatric Septic Shock	RNA microarray			
Multi-center Observational Cohort	N = 98 septic shock			
	N = 32 controls			
Wong et al. 23	Whole blood derived RNA	Endotype A and B	*Grunwell* et al. identified that 11,630 gene probes were differentially expressed comparing endotype A and B. 24	Patients with endotype A who received adjunctive corticosteroids had a fourfold higher rate of ICU mortality relative to patients with endotype who received corticosteroids. 23
Pediatric Septic Shock	Nanostring mRNA quantification		Targeted assessment of *Nrf2* linked genes identified greater downregulation among patients with endotype A, relative to patients with endotype B, suggestive of impaired antioxidant mechanisms	Endotype specific responses will be retrospectively tested in patients enrolled in the Stress Hydrocortisone in Pediatric Septic Shock (SHIPPS, NCT0341398)
	- 96 subclass defining genes, 4 housekeeping genes			
Multi-center Observational Cohort	N = 168 septic shock patients			
	No controls			
2. Yang et al. 25	Whole blood derived RNA	Subclass 1 and 2	Subclass 1 had overexpression of the innate immune arm and repression of the adaptive immune arm. Evidence of higher inflammatory cytokines and endothelial activation in Subclass 1 compared to Subclass 2	Subclass 1 had higher burden of organ failures when compared to Subclass 2. No significant difference in mortality between groups. Did not test heterogeneous response of patients to treatments
Pediatric Septic Shock	Bulk messenger RNA sequencing		Cellular deconvolution analyses suggested decreased CD4- T lymphocytes, B-lymphocytes, and decreased T-cell receptor repertoire	
Secondary analyses of Coagulation and Fibrinolysis in Pediatric Insulin Titration (CAF-PINT) study; ancillary to the Heart and Lung Failure HALF-PINT trial				
	N = 46 septic shock			
	N = 52 mechanically ventilated, non-infected controls			
3. Maslove et al. (2012) 97	Neutrophil derived RNA,	Subtype 1 vs 2	Increased expression of pathogen recognition receptors, interferon and inflammatory cytokine signaling including MAPK and JAK/STAT pathways, B-cell activation, and T-cell activation among subtype 1 vs. 2	No difference in mortality between groups. Higher prevalence of severe sepsis among patients with subtype 1 and a trend toward higher rate of septic shock among patients in subtype 2
	Microarray from 2 prior studies. 98, 99		In addition, increased expression of adrenergic signaling, serotonergic and opiate signaling in subtype 1 vs 2	
	K-means clustering			
	(N = 55 in derivation cohort, 71 in validation cohort)			
4. Davenport et al. 26	Peripheral blood leukocyte derived RNA	Sepsis Response Signature (SRS) 1 and 2	SRS 1 had downregulation of pathogen recognition receptors (PRR) including toll like receptor (TLR), decreased antigen presenting cell (APC) human leukocyte antigen class II (HLA-II) expression, and T-cell exhaustion, in comparison with SRS2 endotype	Patients with SRS1 had a 2 to ~ threefold higher odds of mortality in derivation and validation cohorts, relative to those with SRS2 membership
	Microarray and integration with whole genome genotyping			
Adults with community acquired pneumonia who developed sepsis			Evidence of cis-expression quantitative trait loci (eQTL) associated with endotypes	Antcliffe et al. identified that in secondary analyses of vasopressin, norepinephrine, and steroids (VANISH) trial, patients with the immunocompetent SRS2 signature had an eightfold higher mortality with receipt of randomized hydrocortisone, relative assigned to SRS1. 27
	7 gene-classifier in validation set			
Multi-center observational Cohort			*IL18RAP*, *HIF1A*, *mTOR*, *HSF2* upregulated in SRS1	
Genomic Advances in Sepsis (GAinS) study	N = 265 in derivation cohort		*LAX1*, *TRIM44*, *and DDX24* downregulated in SRS2	
	N = 106 in validation cohort			
5. Sweeney et al. 28	Peripheral blood leukocyte derived RNA, Microarray	Inflammopathic,	Inflammopathic endotype was characterized by pathogen recognition receptor signaling, pro-inflammatory cytokine, IL-1 receptor activity. High percentage of immature neutrophils and lymphopenia	Inflammopathic and coagulopathic endotypes associated with ~ 30% mortality and ~ 25% mortality, while Adaptive endotype had a ~ 8% mortality
		Coagulopathic, and Adaptive		
Pooled meta- analyses of critically ill pediatric and adult patients	N = 700		Coagulopathic endotype characterized by pathways involved in platelet degranulation, heparin- and fibrinogen- binding, and glycosaminoglycan signaling	Prognostic significance among COVID19 patients and Outcomes of Metabolic Resuscitation using Ascorbic acid, Thiamine, and Glucocorticoids in the Early Treatment of Sepsis (ORANGES) trial. 31 Suggestion of heterogeneous response to hydrocortisone, ascorbic acid, and thiamine (HAT) therapy across subclass, however association was not very robust
	500 genes for derivation of endotypes		Adaptive endotype was characterized by activation of antigen presenting cells, T-cell receptor binding and activation	Yao et al. 100 reproduced 3 endotypes in secondary analyses of publicly available datasets. Authors determined prevalence of immune-adaptive prevalent (IA-P) or immune-innate prevalent (IN-P). Tested response to corticosteroids among patients enrolled in VANISH trial
				Use of hydrocortisone was associated with heterogeneity in treatment effect among patients with IA-P but not IN-P
	33 gene-classifier in validation cohorts			
				Patients hospitalized with COVID19, patients with *adaptive* endotype treated with Anakinra were more likely remain in the same endotype. Further, patients with a c*oagulopathic* endotype were half as likely to have severe respiratory failure at day 7, relative to placebo treated patients. 32
	Nanostring based validation in subsequent studies			
6. Scicluna et al. 33	Peripheral blood leukocyte derived RNA, Microarray	Mars 1–4 endotypes	Mars1 endotype was characterized by repression of both innate and adaptive genes including pathogen recognition receptor signaling including TLR and RIG-1 receptors, nuclear factor κB (NFκB1) signaling, antigen presentation, T-helper cell differentiation and receptor signaling	Mars1 endotype was associated with higher hazard of 28-day mortality relative to the other 3 endotypes
Molecular diagnosis and risk stratification (MARS) consortium	N = 306 derivation cohort		Mars2 endotype characterized by increased expression of genes involved in activation of several of these pathways and interleukin-6 (IL-6) signaling, inducible nitric oxide synthase (iNOS), and HIF1A signaling	
Two-center observational cohort	N = 216 validation cohort		Mars3 endotype was associated with activation of pathogen recognition receptor signaling, antigen presentation, IL-4 signaling, differentiation of T-helper cell, natural killer (NK) cells, and B-cell signaling	
			Mars4 endotype was associated with increased expression of genes involved in pattern recognition and cytokine pathways, interferon signaling, RIG1-like receptor and TREM1 signaling, activation of cytotoxic functions of T- and NK-cells	
	N = 265 external validation in GaINS study			
7. Baghela et al. 101	Whole blood RNA	5 endotypes identified, biological features relative to healthy controls and other endotypes	NPS endotype characterized by cellular response to stress, ROS detoxification, and neutrophil degranulation	Only the NPS and INF endotypes had higher mortality relative to other 3 endotypes
	Bulk mRNAseq			
Multi-center prospective observational cohort		Neutrophilic-Suppressive (NPS)	INF endotype characterized by B- and T-cell receptor signaling, and IL-1 signaling	
	N = 348 patients meeting sepsis criteria	Inflammatory/		
4 ERs and 1 ICU		INF	IHD group characterized by neutrophil signaling, IL-1 signaling, cholesterol biosynthesis	
	N = 44 healthy controls			
		Innate-Host-Defense/IHD	IFN endotype characterized by interferon signaling and anti-viral responses	
		Interferon/IFN		
			ADA endotype characterized by cell surface interactions with vascular wall	
		Adaptive/ADA		
8. Yehya et al. (2020). 35	Whole blood RNA	CHOP ARDS Transcriptomic Subtypes (CATS 1–3)	CATS1 was characterized by activation of the adaptive immune response including Th cell differentiation and programmed cell death (PD-1) signaling	CATS1 and 2 had a threefold higher mortality compared to CATS 3. Heterogeneous responses to treatment were not tested
Single-center prospective observational cohort of pediatric ARDS	RNA Microarray		CATS2 showed enrichment for complement activation	
	N = 106		In contrast, CATS3 showed repression of T-cell receptor signaling. Ingenuity pathway analyses revealed activation of upstream regulators of inflammatory cytokines among patients belonging to CATS1 and repression among CATS3 patients	
9. Matthew et al. 102 (2020)	Enriched for lymphocytes	Immunotype 1–3	Immunotype 1 was characterized robust activated CD4 T cells, low circulating follicular T helper cells, activated CD8 effector memory cells (EMRAs)	Immunotype 1 was associated with disease severity
Prospective observational cohort of hospitalized adults with COVID19	High dimensional flow cytometry and longitudinal analyses		Immunotype 2 was characterized by less robust CD4 T cell activation, Tbet + effector CD4 and CD8 T cells, and proliferating memory B cells	Immunotype 2 was not associated with disease severity
	N = 125		Immunotype 3 lacked activated T and B cell responses	Immunotype 3 was associated with low mortality
	And 60 healthy controls and 36 patients with disease recovery			
10. Lopez- Martinez et al. (2023) 103	N = 56	Covid transcriptomic profiles (CTP) 1 and 2	9,700 differentially expressed genes between subclasses	CTP 2 had lower risk of survival to ICU discharge and greater ventilator free days compared to patients with CTP1
	mRNA sequencing, Micro RNA sequencing.		CTP2 was characterized by repression of Type 1 IFN signaling and T and NK cell activity and overexpression of genes involved in B-cell receptor signaling and Treg cell function, relative to CTP1	Receipt of adjuvant steroids resulted in downregulation of T- and B-cell activation, Interleukin production, and activation of JAK/STAT signaling only among patients in CTP1
			Cellular deconvolution analyses revealed CTP2 had a lower mature neutrophil count, higher lymphocytes, naïve B cells, predominance of CD8 + T cells, relative to CTP1	All patients received steroids, so could not assess heterogeneity in treatment effect
			Lower miRNA-145a-5p and miRNA-181-5p among patients with CTP2, relative to CTP1	
11. Calfee et al. 36	Clinical variable, protein biomarkers	Hyper- and hypo-inflammatory phenotypes	Overexpression of innate immune signaling, IL-8 signaling among patients with hyperinflammatory phenotype. Deconvolution analyses suggest predominance of macrophages, dendritic, and natural killer (NK) cells	Hyperinflammatory phenotype associated with higher mortality relative to the hypoinflammatory phenotype
			Overexpression of T-cell receptor signaling, CD28 co-stimulation among those with a hypo-inflammatory phenotype. Deconvolution analyses suggest predominance of T-cells and plasma cells	
Adults with ARDS	Latent class analyses			Interaction between phenotypes and several interventions, including positive end expiratory pressure (PEEP) strategy and fluid management, 39 and simvastatin therapy among patients with ARDS, 40 activated protein c in adults with sepsis, 45 and corticosteroids in COVID19. 41
Secondary analyses of ARMA and ALVEOLI trial, Prospective observational cohorts- EARLI and VALID	*Neyton* et al. subsequently conducted transcriptomic and metagenomic analyses of patients in EARLI and VALID. 49 In separate studies, the authors identified moderate overlap with endotypes of acute pancreatitis. 104			
	*Sinha* et al. conducted transcriptomic of patients with ARDS in secondary analyses of the ROSE trial. 50			
12. Bos et al. 43	Clinical variable, protein biomarkers	Reactive and uninflamed phenotypes	Reactive patients were characterized by overexpression of innate immune signaling, oxidative phosphorylation, antioxidant signaling (Nrf2), cholesterol biosynthesis and by neutrophil activation	Patients with a reactive phenotype had a three-fold higher mortality compared to patients with an uninflamed phenotype
Adults with community acquired pneumonia and sepsis	Clustering analyses		Patients with an uninflamed phenotype had overexpression of MAPK, T-cell lymphocyte signaling and increased apoptosis	
Secondary analyses of MARS consortium	N = 210 patients			
	Peripheral blood leukocyte derived RNA			
	RNA Microarray			

## Gene-expression based endotypes

*Wong* and colleagues first used whole blood RNA microarrays to identify the gene-expression signatures associated with mortality among children with septic shock 19, 20. Subsequently, among the most differentially expressed genes (DEGs), 100 subclass defining genes broadly reflective of the adaptive arm of the host immune response, T-cell function, and glucocorticoid signaling were used to identify patient subclasses through unsupervised clustering analyses 21, 22. Subsequently, two subclasses or endotypes A and B were validated, with patients belonging to *endotype A* being characterized by repression of the adaptive immune and glucocorticoid signaling, relative to patients with *endotype B*. 23 Of note, *endotype A* was associated with a nearly threefold higher odds of mortality 21. Further, patients with *endotype A* who received adjunctive corticosteroids had a fourfold higher rate of ICU mortality compared to *endotype B* who received corticosteroids 23. In follow up studies, *Grunwell* et al. identified that 11,630 gene probes were differentially regulated between patient endotypes A and B 24. Targeted analyses of nuclear factor erythroid-related factor 2 (Nrf2), a transcription factor that regulates expression of anti-oxidant genes, revealed greater downregulation among patients with *endotype A*. More recently among children with septic shock in secondary analyses of the Coagulation and Fibrinolysis in Pediatric Insulin Titration (CAF-PINT) study, *Yang* et al. identified two septic shock subclasses using bulk mRNA sequencing 25. Although the mortality among patients included in this cohort was low, *Subclass 1* had higher burden or organ dysfunction compared to *Subclass 2* and characterized by upregulation of the innate immune and downregulation of the adaptive immune pathways, with evidence of systemic inflammation and endothelial injury based on plasma protein biomarkers. Deconvolution analyses of cellular composition suggested a decrease of CD4 T- and B-lymphocytes, and a lower diversity of T-cell receptors (TCR). 25

Several efforts to identify gene-expression endotypes have been undertaken among adults. *Davenport* et al. conducted peripheral blood leukocyte expression profiles among patients with community acquired pneumonia with sepsis recruited through the U.K. Genomic Advances in Sepsis (GAinS) study 26. They identified two distinct sepsis response signatures (SRS), where *SRS1* was associated with an immune-suppressed phenotype with downregulation of pathogen recognition receptors (PRRs), decreased expression of human leukocyte antigen (HLA) class II on antigen presenting cells (APCs), and T-cell exhaustion, relative to those with *SRS2* endotype. Patients with *SRS1* had a two to threefold higher odds of mortality, relative to those with *SRS2* membership. In secondary analyses of the vasopressin, norepinephrine, and steroids (VANISH) trial, *Antcliffe* et al. identified that patients with the immunocompetent *SRS2* endotype, had an ~ eightfold higher mortality with receipt of randomized hydrocortisone relative to patients designated as SRS1. 27 The authors speculated that among patients with *SRS2*, receipt of corticosteroids may have contributed to greater downregulation of HLA-DR expression contributing to a detrimental response to corticosteroids in this subgroup. The authors took the illuminating step of integrating whole genome genotyping with gene-expression data allowing them to identify expression quantitative trait loci (eQTL) associated with endotypes 26. Interleukin 18 receptor (*IL18RAP)* and chemokine receptor 1 (*CCR1*) were upregulated, while CCR3 was downregulated among patients with *SRS1*. Moreover, hypoxia inducible transcription factors HIF1α (*HIF1A*) and HIF2α (*EPAS1*), mammalian target of rapamycin (mTOR) pathway a key determinant of cell metabolism and fate (Fig. 2), were among the most upregulated genes with evidence of cis-eQTL among patients with *SRS1*.

*Sweeney* et al. pooled pediatric and adult datasets across 14 discovery cohorts comprising 700 patients and utilized meta-clustering approach to identify 3 sepsis endotypes, which they termed *Inflammopathic*, *Coagulopathic*, and *Adaptive*. 28 The *Inflammopathic* subclass was characterized by pathogen recognition receptor signaling, pro-inflammatory cytokine signaling and complement activation and had ~ 30% mortality. The *Coagulopathic* subclass was characterized by pathways involved in platelet degranulation, glycosaminoglycan-, heparin-, and fibrinogen-binding, and broadly reflective of coagulation cascades and had ~ 25% mortality. Finally, the *Adaptive* subclass was characterized by activation of pathways related to antigen presentation activity, T-cell receptor binding, activation of lymphocytes, and had a mortality of ~ 8%. This group deployed a 33-gene classifier to assign endotypes which has demonstrated reproducibility in when applied in secondary analyses of observational cohort of patients with surgical sepsis 29, COVID19 patients 30, and heterogeneous treatment effects in a randomized trial assessing Outcomes of Metabolic Resuscitation using Ascorbic acid, Thiamine, and Glucocorticoids in the Early Treatment of Sepsis (ORANGES) trial 31, and most recently interleukin-1 receptor antagonist in the SAVE-MORE trial among patients with COVID19. 32

*Scicluna* et al. identified 4 endotypes among adults with sepsis enrolled in the prospective observational cohort by the molecular diagnosis and risk stratification (MARS) consortium based in the Netherlands 33. The *Mars1* endotype was associated with higher hazard of mortality relative to the other 3 endotypes, and characterized by repression of both innate and adaptive genes including PRR signaling, nuclear factor κB (NFκB) signaling, APC signaling, T-helper cell differentiation and TCR signaling (Figs. 2 and 3). Clinical outcomes were similar among Mars 2–4 endotypes. This group developed a 140-gene classifier which demonstrated reproducibility. Of note, Mars3 endotype showed a significant association with the immunocompetent *SRS2* and *Adaptive* endotypes, described previously. 26, 34

Transcriptomic studies among patients withs ARDS focused on identifying subclasses have been limited 35. *Yehya* et al. identified 3 subclasses named the Children’s Hospital of Philadelphia ARDS Transcriptomic Subtypes (CATS) among 96 pediatric patients using whole blood mRNA. *CATS1* subclass was characterized by persistent hypoxemia and ~ 30% mortality. Nearly half of the patients assigned to *CATS2* had an immunocompromised status but rapidly resolved hypoxemia and ~ 25% mortality. Patients among CATS3 had the lowest mortality ~ 8%. *CATS1* was characterized by T helper cell differentiation and programmed cell death (PD-1) signaling. *CATS2* showed enrichment for complement activation and *CATS3* had repression of TCR signaling. Pathway analyses suggested activation of upstream regulators of inflammatory cytokines among *CATS1* and repression among *CATS3* patients. Receipt of and response to corticosteroids were not reported in this cohort.

## Latent class or clustering based phenotypes

In seminal work, *Calfee* et al. leveraged latent class analyses using clinical, laboratory, and biomarker data to identify two subphenotypes of ARDS among patients recruited through the ARDS network trials 36. Patients with higher probability of membership among phenotype 2, identified as *hyperinflammatory* based on higher plasma concentrations of proinflammatory cytokines and markers of endothelial activation including IL-6, IL-8, soluble tumor necrosis factor receptor-1 (sTNFr1) and plasminogen activating inhibitor-1 (PAI-I) and lower concentrations of protein C, had a higher prevalence of sepsis and worse clinical outcomes relative to those without this phenotype. *Dahmer* et al. showed reproducibility and prognostic utility of this approach among children with ARDS in secondary analyses of the Randomized Evaluation of Sedation Titration for Respiratory Failure (RESTORE) trial 37; similar findings have been made in other observational cohorts 38. Of note, latent profile phenotypes have demonstrated interaction with several interventions 36, 39 and simvastatin therapy in the HARP-2 trial, on clinical outcomes 40. Using a different set of clinical variables and fewer biomarker inputs, *Sinha *et al*.* identified two phenotypes among adults with COVID19 with treatment with corticosteroids being associated with improved survival among those with *hyperinflammatory* phenotype and higher mortality among those with a *hypoinflammatory* phenotype. 41

Studies conducted by investigators in the MARS consortium have reproduced the findings of Calfee and colleagues. *Bos* et al. used cluster analyses to identify a *reactive* and *uninflamed* phenotype, with worse outcomes observed among those with a *reactive* phenotype 42. Assessment of leukocyte-expression profiles revealed genes were broadly representative of innate immune signaling and neutrophil activation 43. Pathway analyses revealed that that oxidative phosphorylation pathways, cholesterol biosynthesis, and antioxidant signaling through Nrf2 were upregulated among patients with a *reactive* phenotype (Fig. 2). In contrast mitogen activated protein kinase (MAPK) pathways, T-cell signaling, and apoptosis were upregulated among patients with an *uninflamed* phenotype. *Heijnen* et al. identified that these phenotypes maintained their biological distinctiveness among mechanically ventilated patients irrespective of whether they met ARDS criteria, and showed similarity with outputs of latent class analyses. 44

S*inha* and colleagues recently published on molecular phenotypes among adults with sepsis 45. In two prospective observational cohorts, the authors established the prognostic utility of latent class phenotypes among adults with sepsis with high concordance with phenotypes among patients with ARDS. When re-analyzing data from the PROWESS-SHOCK study 46, the authors noted a differential response to recombinant human activated protein C between phenotypes, with patients with a greater probability of a *hyperinflammatory* phenotype demonstrating a beneficial effect with receipt of therapy. Of note, there was no evidence of differential responses among phenotypes based on vasopressor choice when re-examining results of the Vasopressin in Septic Shock Trial (VASST); these results are concordant with findings with similar studies. 47, 48

Recent studies by *Neyton* et al. among adults with sepsis revealed overexpression of genes representative of the innate arm among those with a *hyperinflammatory* phenotype and those of the adaptive arm among patients with an *hypoinflammatory* phenotype 49. Patients in the VANISH study were subsequently assigned to the phenotypes based on a gene-expression classifier and the authors identified that patients with *hypoinflammatory* phenotype overlapped with the immune competent *SRS2* endotype and demonstrated higher mortality with receipt of hydrocortisone compared to placebo. Such an effect was not noted among patients with the *hyperinflammatory* phenotype. *Sinha* et al. conducted transcriptomic analyses among a subset of patients assigned to ARDS phenotypes enrolled in the re-evaluation of the systemic early neuromuscular blockade (ROSE) trial yielding similar results 50. Of note, temporal shifts in gene-expression identified among subclasses influenced by patient survival, rather than phenotypic assignment, highlighting the dynamic nature of critical illness and molecular complexities involved.

Finally*, Bhatraju* et al. used latent class analyses using biomarkers of inflammation and endothelial activation to identify two subphenotypes of AKI- *AKI-SP1* and *AKI-SP2* in independent discovery and validation cohorts 51. The *AKI-SP2* subgroup characterized by elevated Angiopoietin-2/Angiopoietin-1 ratio had a ~ 2.5-fold higher risk of 28-day mortality and increased risk of renal non-recovery, relative to patients with *AKI-SP1*. In secondary analyses of the VASST trial, patients with AKI-SP1 had a differential response to vasopressors with lower 28- and 90-day mortality among those receiving vasopressin compared to norepinephrine. However, this was not the case with the high-risk AKI-SP2 subphenotype. Studies by *Wiersema* et al. in secondary analyses of the Finnish AKI (FINNAKI) study yielded similar subclasses with prognostic utility 52. It is worth noting that the biomarkers used for phenotyping patients are not specific to AKI and generalizable across critical illness syndromes.

## Corroboration in pre-clinical models

Few studies have attempted to develop animal models capable of recapitulating biological heterogeneity noted among critically ill patients. In cecal ligation puncture (CLP) model of sepsis, *Wong* et al. identified that murine analogues of pediatric sepsis mortality risk biomarkers (PERSEVERE) could be utilized to risk-stratify experimental mice 53. In an analogous approach, *Seymour* et al. have used biotelemetry-enhanced CLP coupled with latent class analyses to identify two phenotypes among experimental mice, with one of the classes demonstrated shorter time to deterioration and greater concentrations of biomarkers of systemic inflammation 54. Interestingly, in a subset of mice that were randomized to receive immediate vs. delayed fluid resuscitation and antibiotics; only the sicker subset demonstrated improvements with therapy. To model complexity, investigator groups have utilized large animal models. *Millar* et al. have developed an ovine model of ARDS using a two-hit approach 55. Importantly, animals received intensive care. A priori clustering analysis was used to identify two phenotypes, with one of the phenotypes characterized by higher plasma concentrations of IL-6, IL-8, and IL-10 and manifesting similar features as ARDS patients with a *hyperinflammatory* phenotype. Targeted study of gene-expression revealed that this phenotype was characterized by overexpression of neutrophil genes also implicated in the human host response 56. In subsequent studies the group identified that randomization to corticosteroids was associated with a benefit only among the ovine *hyperinflammatory* group 57. A few important limitations worth considering including the fact that experimental approaches described may merely account for variance in the procedures to induce critical illness in animals. More importantly, models either use animals with a homogenous genetic background or those with knockout of select genes. While the latter are useful to understand the mechanism of individual genes, they do not reflect the tremendous genetic diversity among patients that vitally contribute to patient-, organ-, and cell-specific heterogeneity, thus inherently limiting their translational potential. Lastly, to the best our knowledge, no in vitro models have thus far been developed to recapitulate genetic heterogeneity nor the organotypic responses observed among critically ill patients –an area of interest emphasized by the U.S National Advisory General Medical Sciences Council (NAGMSC) working group on sepsis. 58

## Current translational gaps

The identification of subclasses with prognostic and predictive relevance is a commendable feat. Yet, we remain in the relative infancy of our understanding of subclass-specific disease mechanisms. As an analogy, the endotypes and phenotypes described thus far may serve as lenses of a compound microscope through which we are able to begin to grasp a more granular understanding of the mechanisms underlying critically ill patients. However, there is an urgent need for implementation of advanced scientific tools and disease models to open new vistas for the discovery of biological drivers underlying subclasses. Further, it remains possible that there exist relevant yet undiscovered biological pathways that are not restricted by the current framework for critical illness subclasses. Accordingly, development of both top-down and bottom-up approaches, which consider current subclasses, are necessary to better understand causal biological pathways underlying critical illness.

## Towards consensus endophenotypes

**Fig. 4:**
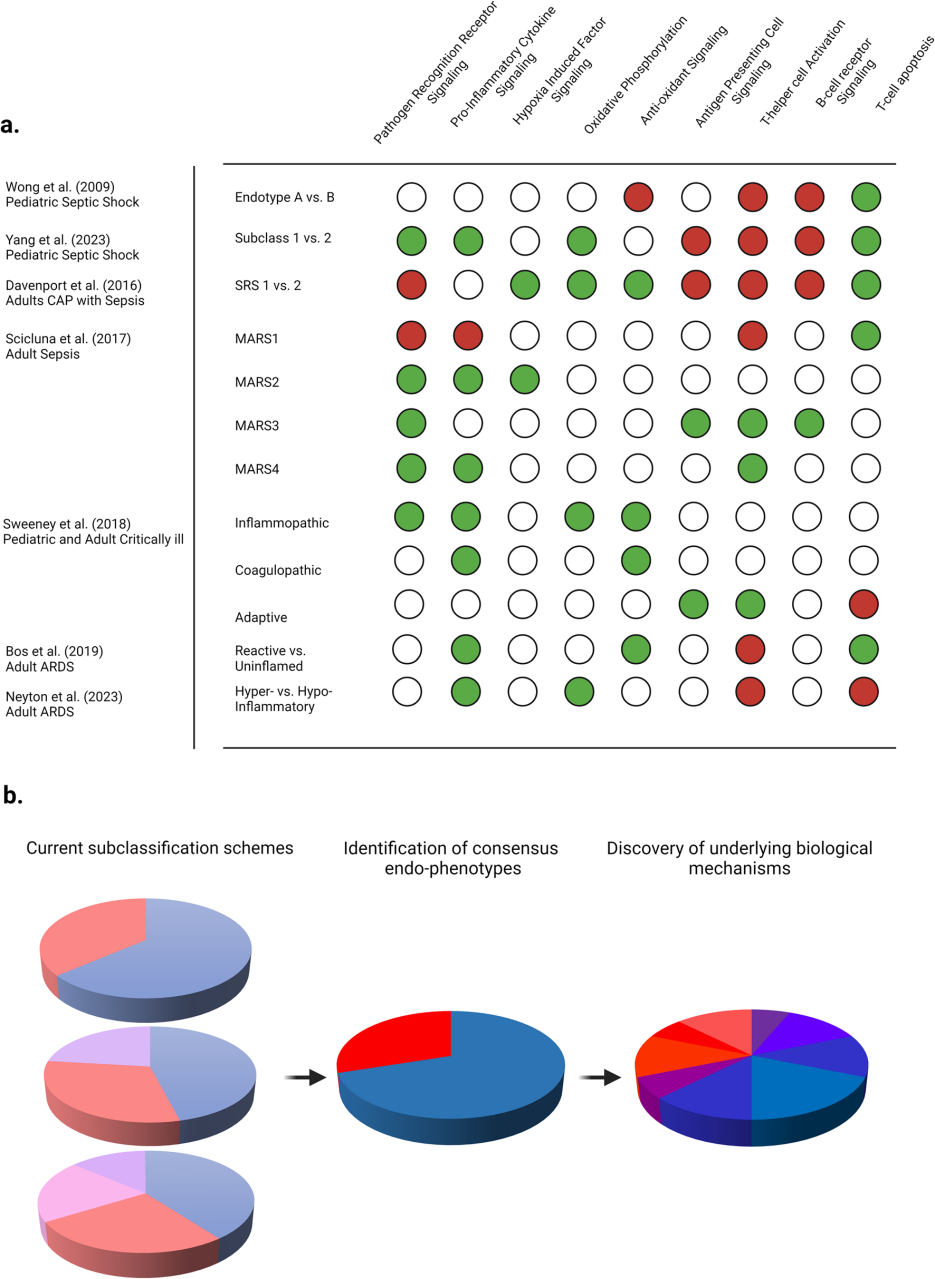
**a** Similarities and differences in activation of key signaling pathways based on gene-expression data among critical illness subclasses according to scheme used. Key biological facets shown include pathogen recognition receptor signaling, pro-inflammatory cytokine signaling, hypoxia induced factor signaling, oxidative phosphorylation, antioxidant signaling, antigen-presenting cell signaling, T-helper lymphocyte activation, B-lymphocyte receptor signaling, and T-lymphocyte apoptosis. The green dots indicate activation while the red dots represent inactivation, as detailed in the referenced articles. The studies are grouped based on age and critical illness syndromes including sepsis, septic shock, and acute respiratory distress syndrome as detailed in the y-axis.** b** Conceptual overview of (1) current critical illness subclassification schemes sampling the proverbial 'biological pie' in varying slices and depth, (2) the need to move toward consensus critical illness endophenotypes through which the underlying molecular mechanisms can be unraveled, and (3) identification of subclass-specific molecular features or treatable traits that may be amenable to targeted therapeutic intervention

In Fig. 4a, activation (green) and inactivation (red) of intra-cellular signaling pathways identified through gene expression studies are shown according to critical illness syndrome and the subclassification scheme used; evidently some approaches are more congruent than others. In a recent study *van Amstel* et al. compared class outputs of several critical illness subtyping schemes within the same set of patients and identified relatively low to moderate overlap between clinical, biomarker, and transcriptomic data-based approaches 59. Furthermore, several studies have identified that integrated subclassification approaches, for example those that combine biomarker and transcriptomic approaches, may be more informative than using a single approach alone [59–61. Moreover, it is established that nearly half of the patients switch between gene-expression endotypes assigned on day 1 by day 3 among adults 62 and children 63. In contrast, limited data among adult ARDS phenotypes suggest that ~ 95% of patients maintained original class assignment. It remains possible that given the greater molecular depth afforded by gene-expression, in comparison with clinico-biomarker approaches, that subclass switching is more readily apparent when using the former approach.

Given these challenges, achieving consensus approaches to identify clinically relevant, biological informative, and temporally characterized subclasses agnostic of underlying syndromic assignments and host developmental age is crucial, as illustrated in Fig. 4b. Towards this end, efforts towards identifying consensus endotypes are underway through the SUBtyping in SePsis And Critical illnEss (SUBSPACE) consortium – a partnership between academia and industry to identify consensus gene-expression endotypes among critically ill adults and children. Similar outputs may be expected from the recently formed ARDS, Pneumonia, Sepsis (APS) consortium—funded by the U.S National Institute of Health. Forward thinking adaptive study designs as with the Precision medicine Adaptive Network platform Trial in Hypoxemic acutE respiratory failuRe (PANTHER) 64 and the treatable traits in acute critical illness (TRAITS) study 65 are likely to further inform the biology of critical illness subclasses. Finally, temporally sampled biospecimens from both prospective cohort and randomized clinical trials before and after intervention hold tremendous potential to inform efficacy of biological response to therapies among critical illness subclasses 66. Several challenges are worth considering. (1) Clinical phenotyping alone in the absence of biologically informative inputs may limit reproducibility of subclasses. (2) Inclusion of appropriate non-critically ill controls is essential, especially to gain insights into critically ill patients who have relatively repression of protein or genes in comparison with the sickest subset of patients. (3) Sampling the host response at multiple-time points is necessary to characterize longitudinal disease trajectories 67 among patients with emphasis on understanding the molecular drivers that predispose patients toward these trajectories. (4) Given the statistical power necessary, investment in federated databases that allow for pooled analyses of a large sample size of patients with rich clinical annotations will be needed. (5) Finally, prioritization of subtyping methods, especially in the absence of obvious overlaps between class outputs, with preferential weighting of approaches that yield reproducible subclasses with demonstrated heterogeneity in treatment effect to interventions and therapies will be crucial.

## Multi-compartment sampling of the host response

Progress and challenges in profiling the dysregulated immune response in critical illness has been excellently summarized elsewhere 68. A major limitation has been that most studies among critically ill patients have focused on the peripheral blood compartment in isolation and not considered the compartmentalized effects of the host response among patients 69. *Heijnen* et al. profiled a limited set of inflammatory cytokines and lung microbiota using mini-BAL specimens among 26 patients sampled among patients with *reactive* and *uninflamed* phenotypes identified based on blood biomarkers 44. Although limited by sample size, the authors did not find significant differences between phenotypes leading them to conclude that the blood biomarker-based designation of phenotypes may not inform the alveolar compartment. Given the potential for both concordant and discordant effects of therapies on systemic and local pro- and anti-inflammatory responses, concerted efforts are necessary to sample multiple compartments among patients. To this end, some groups have had success with use of minimally invasive sampling to sample the tissue-level host response. 70, 71

## Multi-omic profiling to unravel subclass-specific biology

As opposed to Mendelian diseases that arise from very rare genetic variants with a high penetrance, critical illnesses are inherently complex with several relatively common genetic variants with low penetrance acting in concert to influence patient outcomes. This polygenic nature of critical illness 72 has meant that while innumerable studies have characterized the roles of individual genes and related variants among critically ill patients, we are yet to identify approaches to synthesize this complexity and tailor interventions based on knowledge of patient genotype or haplotype. Thus, systematic efforts to identify the influence of the host genome on subclass-specific biology is imperative. Further, given that sampling different omic layers provides orthogonal evidence, integrated multi-omic analyses are likely to provide greater confidence in subclass-specific biological pathways identified. Lastly, few small-scale studies have evaluated epigenome wide shifts among critically ill patients 73, 74. Given the increasing appreciation of the role of epigenetic regulation in the coordination of orchestrated transcriptomic responses 75, simultaneous study of epigenome and transcriptome both at a patient- and cellular-levels may allow for identification of gene-regulatory networks and drivers of subclass-specific biology.

## Dawn of single-cell omics in critical illness pathobiology

Advances in single cell RNA sequencing (scRNAseq) approaches have increasingly allowed for a greater understanding of molecular changes among cellular subsets among critically ill patients. *Reyes* et al. published a comprehensive atlas of the immune landscape among critically ill adults and identified a unique CD14 + monocyte state specific to septic patients 76. Notably, *Kwok* et al. recently published a whole-blood multi-omic atlas and identified that developing neutrophils and emergency granulopoiesis were key drivers of an extreme endotype among critically ill adults with sepsis 77. Lastly, numerous well designed multi-omics studies 78 including those by the COVID-19 Multi-Omic Blood Atlas (COMBAT) consortium 79 have shed light on organ- and cell-specific molecular perturbations among critically ill patients. Integration of such advanced sequencing technologies with conventional approaches to endophenotype patients holds potential to inform subclass-specific biology. It remains yet unknown whether such knowledge can be harnessed to tailor cell-subset specific precision therapies.

## Understanding host–pathogen and microbiome interactions

Advances in scientific methods have enhanced the ability to identify pathogens which contribute to critical illness but often not captured by conventional culture techniques. Using 16s ribosomal RNA sequencing *Dickson* et al. identified that in humans and experimental sepsis, gut-specific bacteria were more common and abundant in bronchoalveolar lavage fluid and correlated with degree of systemic inflammation, relative to healthy controls 80. Further alveolar inflammation was correlated with perturbations in the lung microbiome. Subsequent studies have identified that among critically ill patients enrolled through the Biomarker analyses in Septic ICU patients (BASIC) - a subset of the MARS consortium, patients with increased lung bacterial burden had fewer ventilator free days and associated with development of ARDS. Of note, the presence of gut-associated bacteria including *Lachanospiraceae* and *Enterobacteriaceae* in the lung were predictive of the worst outcomes. 81

*Kalantar* et al. deployed metagenomic next generation sequencing (mNGS) to distinguish patients with and without critical illness due to infectious etiologies and enhance sepsis diagnosis 82. More recently, patients with a *hyperinflammatory* phenotype were observed to have a greater abundance of bacterial reads particularly *Enterobacteriaceae* species identified through mNGS relative to those with a *hypoinflammatory* phenotype, substantiating findings that patients with a *hyperinflammatory* phenotype were more likely to be bacteremic based on conventional results of culture 45. In summary, the interaction between host genetics, pathogen and antibiotic exposure, and alterations in host microbiome at a systems-level remains relatively understudied. Understanding their contribution to disease progression among critical illness subclasses may hold potential to inform targeted interventions.

## Urgent need for human relevant disease models

Current in vitro models based largely on culture of monolayers of cells are reductionist and limited in their ability to reveal organ-specific and compartmentalized responses in critical illness. Recent advances in the field of regenerative medicine hold potential to begin to address these challenges. Human induced pluripotent stem cells (hiPSCs), reprogrammed from somatic cells including PBMCs, are replenishable sources of cells which can be differentiated into any cell type 83. Human iPSCs have been used to develop sophisticated in vitro 3-D organoid models including multi-compartment models 84 and used for therapeutic drug screening and monitoring. Importantly, hiPSCs have been shown to recapitulate functional, phenotypic, and transcriptomic responses of primary—both circulating and tissue-resident cells [85–87. Several studies have used to study cellular responses to pathogenic agents, [88–90 including a recent study where the investigators treated vascularized organoids with plasma from COVID19 patients to recapitulate in vivo responses 91. Further, novel gene-editing tools including CRISPR-cas9 hold potential to develop in vitro models by efficiently knocking down or out one or more genes simultaneously and accelerate mechanistic studies of candidate targets identified through reverse translational approaches. Lastly, patient-specific iPSCs have been used for precision disease modeling in a variety of chronic conditions with monogenic or polygenic inheritance patterns [92–95.Although untested, it is possible that patient-specific iPSCs derived from critically ill patients by capturing human genetic diversity may facilitate the development of *avatars* for precision medicine to study critical illness subclass-specific biology.

## Multi-modal approaches to inform patient care

**Fig. 5:**
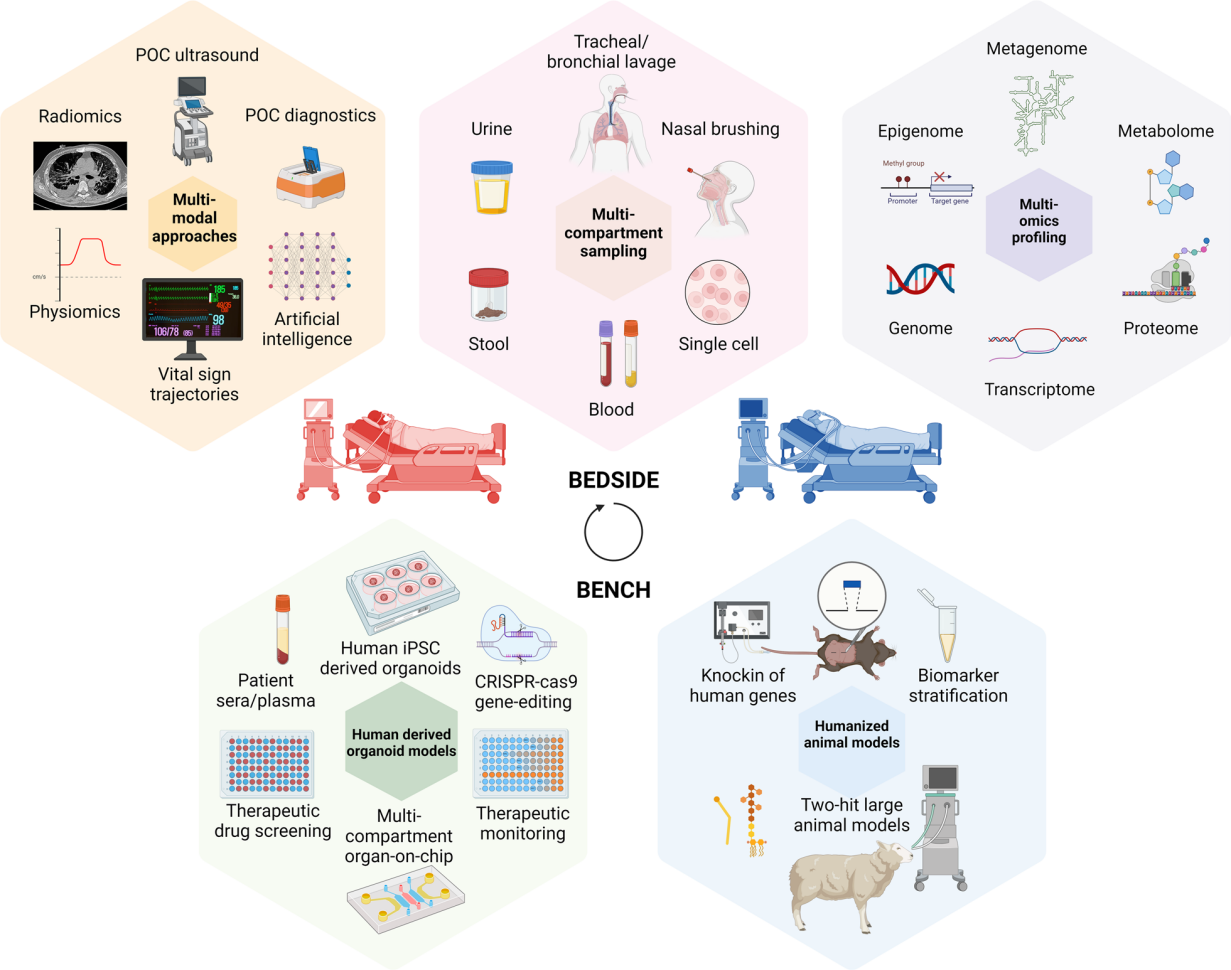
Overview of bedside-to bench-to bedside approaches necessary to better understand drivers of biological mechanisms underlying critical illness subclasses and inform patient care. Starting from center top illustration in a clockwise direction **A.** Multi-compartment sampling among critically ill patients including whole blood including single cell suspensions, nasal brushings, humidified moisture exchange (HME) filter, tracheal aspirate, broncho-alveolar lavage, urine, and stool (in light pink), **B.** Multi-omics profiling including epigenome, genome, transcriptome, metabolome, and metagenome sequencing of biospecimens, including at single-cell resolution where feasible (in light purple). Exploratory multi-omic-data generated from human biospecimens require validation and experimental testing to gain mechanistic insights, necessitating biologically relevant disease models. **C.** Humanized animal models to capture biological heterogeneity including use of ‘knock in’ of human genes in place of murine analogues and biomarker-based stratification or sub-classification of experimental animals and may be used to recapitulate biology of human critical illness phenotypes in vivo. In addition, large animal models subject to environmental factors such as invasive mechanical ventilation are needed to improve disease modeling and testing efficacy of interventions. **D**. Human derived organoid models including those healthy donor and patient-specific induced pluripotent stem cells (iPSCs) treated with sera/plasma from critically ill patients may be potentially used to recapitulate biology of patient endotypes in vitro. The use of CRISPR-cas9 gene-editing technology is anticipated to facilitate a more rapid understanding of genes identified in cell- and organ-specific responses in critical illness. Moreover, these human relevant models can be used to understand compartment-specific responses in vitro, facilitate therapeutic drug screening, and drug monitoring. Importantly, concerted efforts are necessary to integrate biological data with other data streams back at the bedside. **E.** Integration of multi-modal data including vital sign trajectories, physiological and radiological data, point of care ultrasound, although not directly biological informative, are essential to integrate with point of care diagnostic assays that provide biological insights. Built-in artificial intelligence (AI) systems will be essential in the future to synthesize data streams and provide decision-making support to treating clinicians

The wealth of biological information generated among critically ill patients is likely to inundate human capacity to meaningfully process and interpret the data, thus necessitating the deployment of supervised and unsupervised machine learning approaches to distinguish signal from noise. Moreover, as illustrated in Fig. 5, future intensive care practitioners will be required to process a wealth of multi-modal data including electronic and biological data, the latter generated through rapid molecular assays. As such the role of artificial intelligence (AI) for data integration, synthesis, and decision-support cannot be overemphasized 96. Thus, concurrent advances are necessary to bridge the divide between systems-biology based understanding of patient-level pathobiology and clinical informatics to inform patient care at the bedside.

## Conclusions

Advances in precision medicine approaches of the previous two decades have led to the identification of numerous subclassification schemes among critically ill patients. Given the disparate outputs of current subclassification schemes, progress toward identifying consensus endophenotypes is necessary. Furthermore, we remain at the very beginning of the path to identifying treatable traits underlying patient subclasses. Deployment of advanced ‘omic’ technologies and analytic tools are likely to yield subclass-specific candidate biological pathways for hypotheses testing. Moreover, development of robust human relevant disease models are necessary to disentangle mechanistic basis of molecular drivers of critical illness subclasses. Embracing such multi-dimensional approaches may help catalyze the translation of subclass-specific insights into targeted and efficacious interventions and help deliver on the promise of precision critical care medicine to improve patient outcomes.

## Data Availability

Not applicable.
